# Supplementary material for: Correlative Light and Electron Microscopy (CLEM) Analysis of Nuclear Reorganization Induced by Clustered DNA Damage Upon Charged Particle Irradiation
Source: Int J Mol Sci. 2020 Mar 11;21(6):1911. doi: 10.3390/ijms21061911 (PMC7139895; doi:10.3390/ijms21061911)
Supplement: Supplementary file 1 [file ijms-21-01911-s001.zip › SupplementalMovies_Description_.pdf]

Supplemental Movie SM1

- Stack of TEM images recorded from 31 serial thin sections of irradiated U2OS cell stained with ChromEMT. Selected frames of this stack are shown in Figure 3 and Figure S3. Scale bar corresponds to 5 μm.

Supplemental Movie SM2

- Tomographic reconstruction of chromatin organisation. The movie shows 2nm tomographic slices through the 250 nm thick section of cell nucleus of U2OS cell stained with ChromEMT . Notice the absence of distinguishable differences in chromatin organisation within the IRIF (outlined in white) and outside. Selected frames are shown in Figure 5. Scale bar corresponds to 500 nm.
