# Supplementary material for: Correlative Light and Electron Microscopy (CLEM) Analysis of Nuclear Reorganization Induced by Clustered DNA Damage Upon Charged Particle Irradiation
Source: Int J Mol Sci. 2020 Mar 11;21(6):1911. doi: 10.3390/ijms21061911 (PMC7139895; doi:10.3390/ijms21061911)
Supplement: Supplementary file 1 [file ijms-21-01911-s001.zip › Supplemental_Figures.pdf]

Supplemental Figure S1

- **Granules within LDAs in UA stained sections:**
- In some LDAs electron dense granules could be clearly visualised. Most of them appear within or at the direct boarder of the LDAs. They could be similarly observed independent of fixation and embedding applied both at 1 or 5h post-irradiation as well as after irradiation with different ions. (A): Fe-ions, PFA fixation 5h after irradiation, LR White embedding; (B): C-ions, GA 1h after irradiation, Epon embedding; (C): After targeted irradiation with C-ions at the microprobe, GA 1h after fixation, Epon-embedding. Scale bar: A-C 1 µm

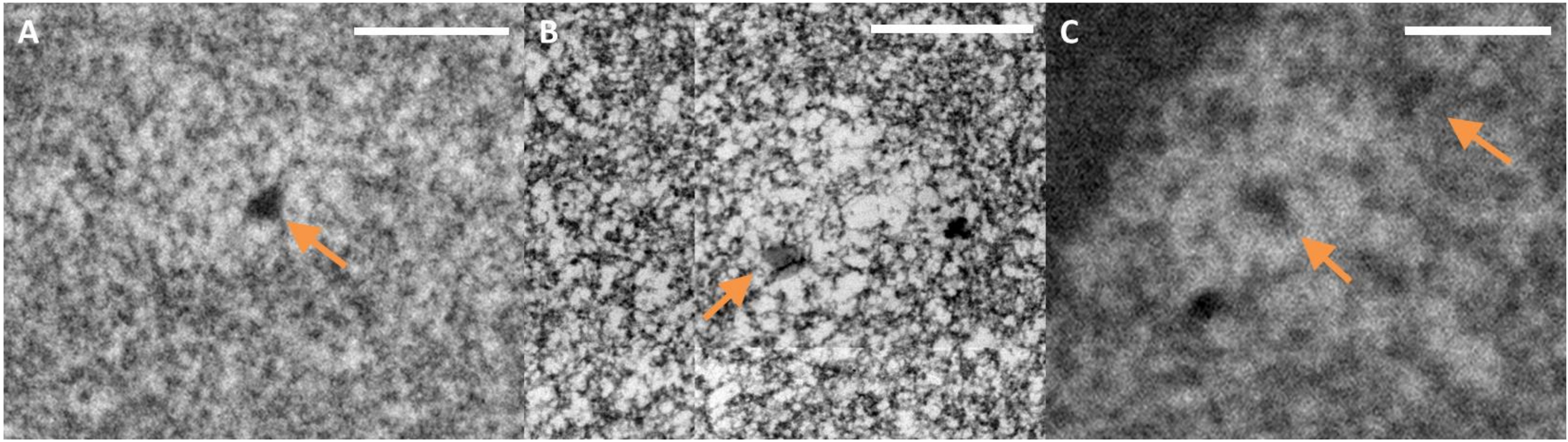
A
B
C

Supplemental Figure 2

- **Comparison of UA and OA-B stained sections of the same cell nucleus:**
- NIH/3T3 nucleus after targeted irradiation with C-ions at the microprobe (cross pattern, fixed 1h after irradiation). (A): Transmission light microscope image obtained at the microprobe for targeting. Red cross indicates the target point and the irradiation pattern. (B): TEM image of a 100 nm section stained with UA. LDAs are outlined with red boxes. (C) TEM image of a section previous to the one shown in (B) and stained with OA-B. It shows no visible LDAs. Same nucleoli (labelled with N) appear highly dense in (B), but are recognised as “empty” areas after DNA-specific OA-B staining (C). In contrary, OA-B stain results in a high contrast of heterochromatin clusters (C, arrows) that are not discernible after the unspecific staining with UA (B). All scale bars: 5 µm

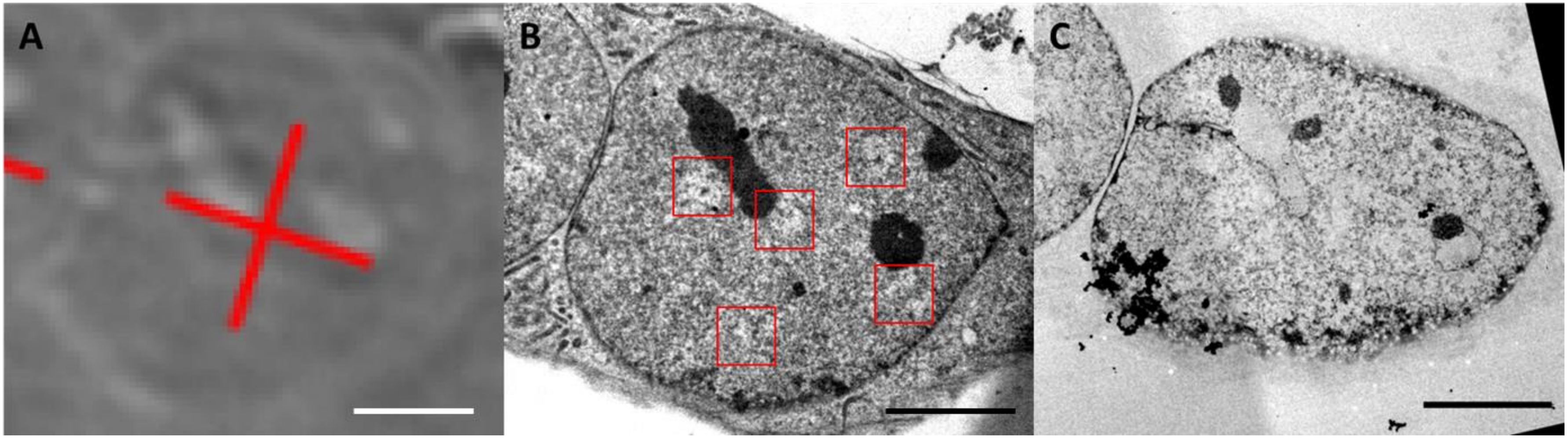

A
B
C

Supplemental Figure S3

- **Localisation of IRIFs by correlation of TEM images of serial sections stained with ChromEMT and confocal FM slices of the same cell:**
- TEM images of serial thin sections after ChromEM staining (first row) and confocal fluorescence images of DRAQ5 (magenta) and 53BP1-GFP (green) (second row) 1h after irradiation with carbon ions. Through the whole nucleus the distribution and shape of the HC are comparable between TEM images (EC visible as dark areas within the nucleus) and FM images (bright magenta coloured signals). Furthermore the shape of the nucleus itself appears very similar indicating the absence of major fixation and embedding related rearrangements. All scale bars: 5 µm

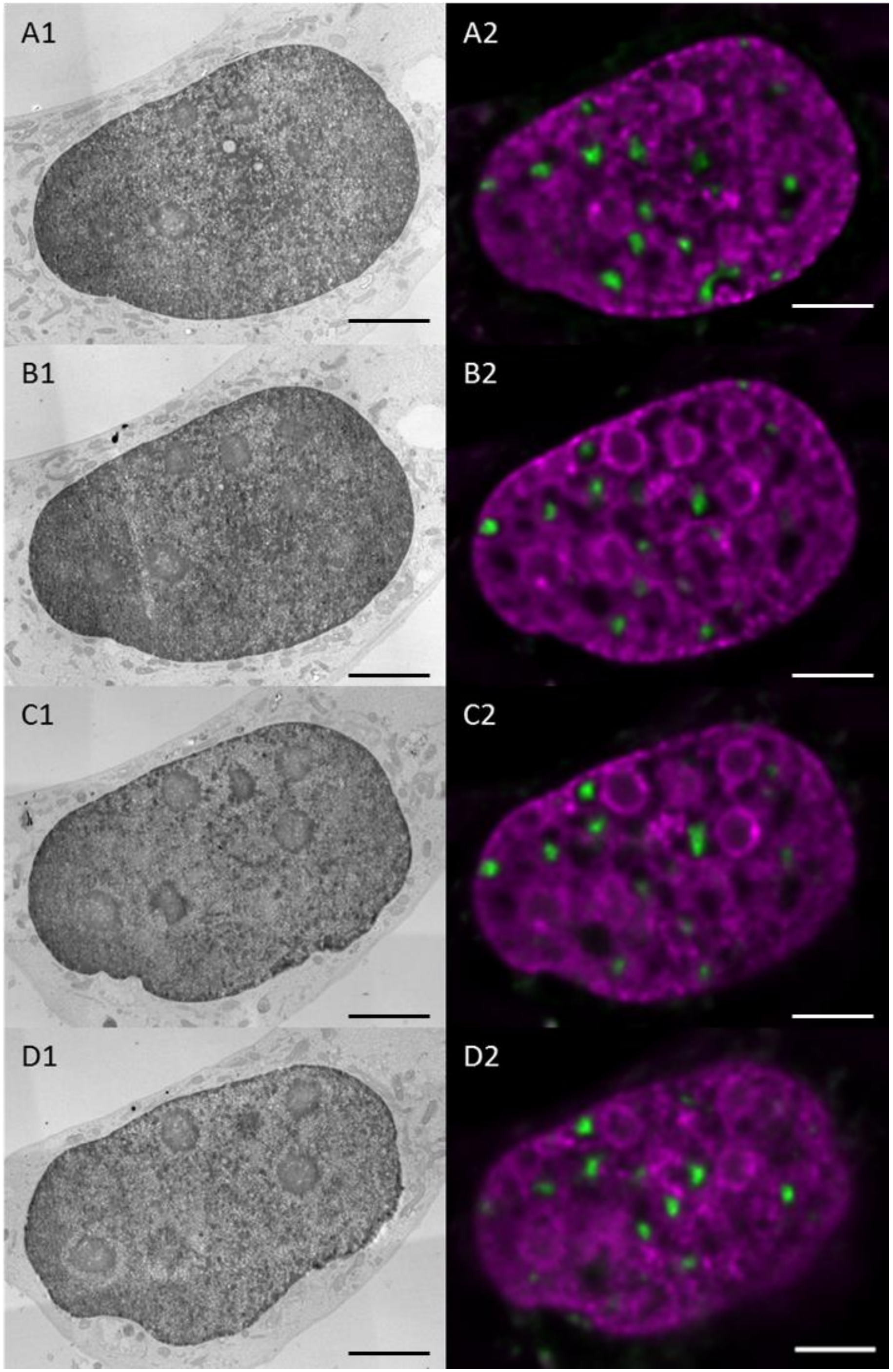
A1
A2
B1
B2
C1
C2
D1
D2

Supplemental Figure S4

- **Targeting areas for tomography:**
- In the FM image (A) an irradiated (C-ions, 1h, 53BP1 IRIF, white box) and a non-irradiated area were defined (dashed white box). These areas were correlated with the TEM image (B). HC (white circles) serves as fiducial marker for subsequent tomography (C and D showing the projection). (C) corresponds to area of white box (irradiated) and (D) to dashed white box (non irradiated). Scale bar: A&B 5 µm, C&D 500 nm

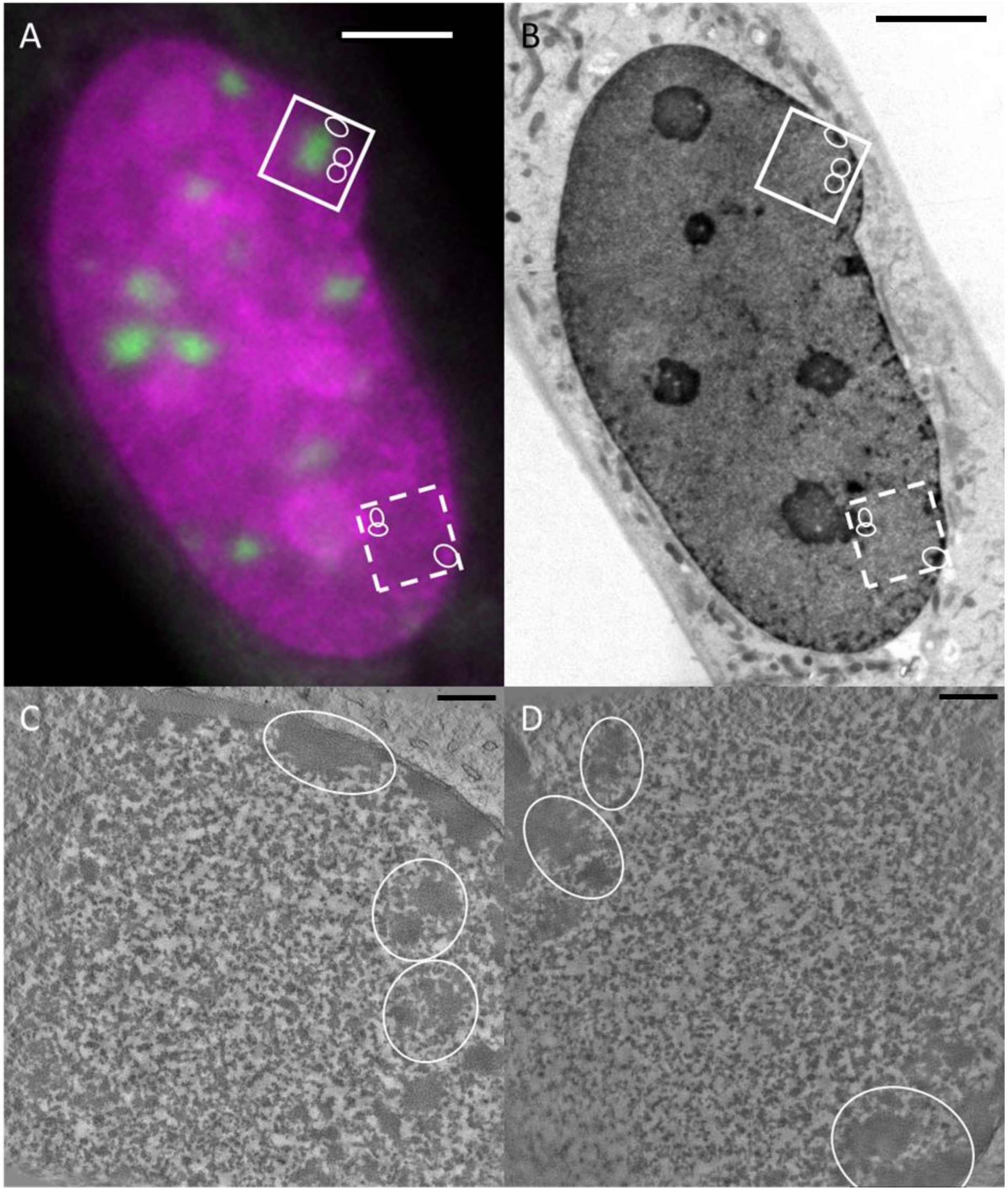
A
B
C
D

Supplemental Figure S5

- **ChromEMT of non-irradiated area:**
- *Gallery of representative 2 nm thick tomographic slices extracted from a dual axis tomographic reconstruction of 250 thick section of a non-irradiated area from the same cell as Figure 5. Mask for analyzed area (white outline) was created from IRIF contour and randomly placed. All scale bars: 500 nm*

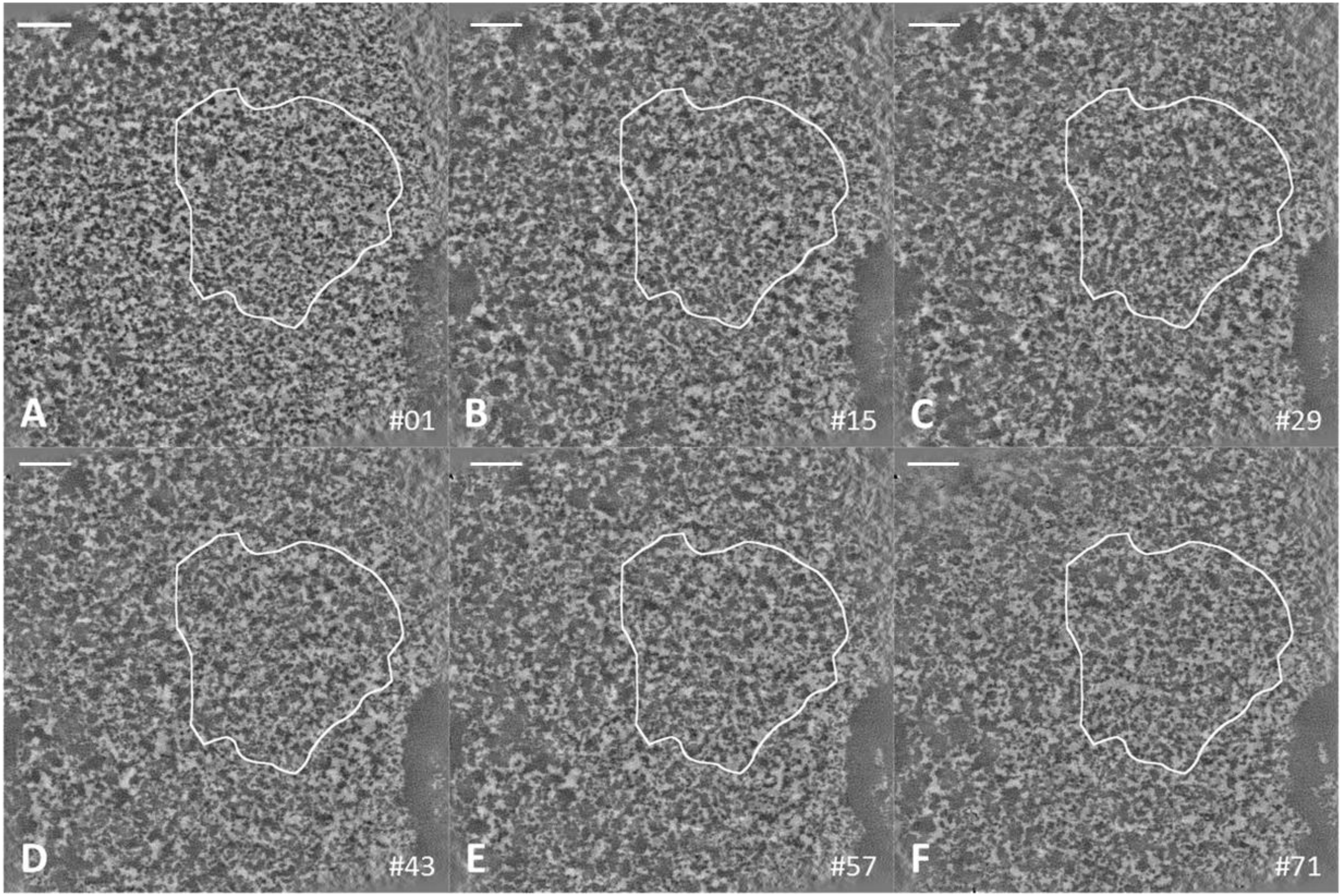
A
#01
B
#15
C
#29
D
#43
E
#57
F
#71

Supplemental Figure S6

- **Defining area for analysing of HC:**
- For the heterochromatin analysis of the tomograms a mask was defined comprising a similar volume as of IRIF/EC analysis, but only including HC. (A) inverted EMT image, (B) generated mask and (C) selected volume for fibre analysis. Scale bar: 500 nm

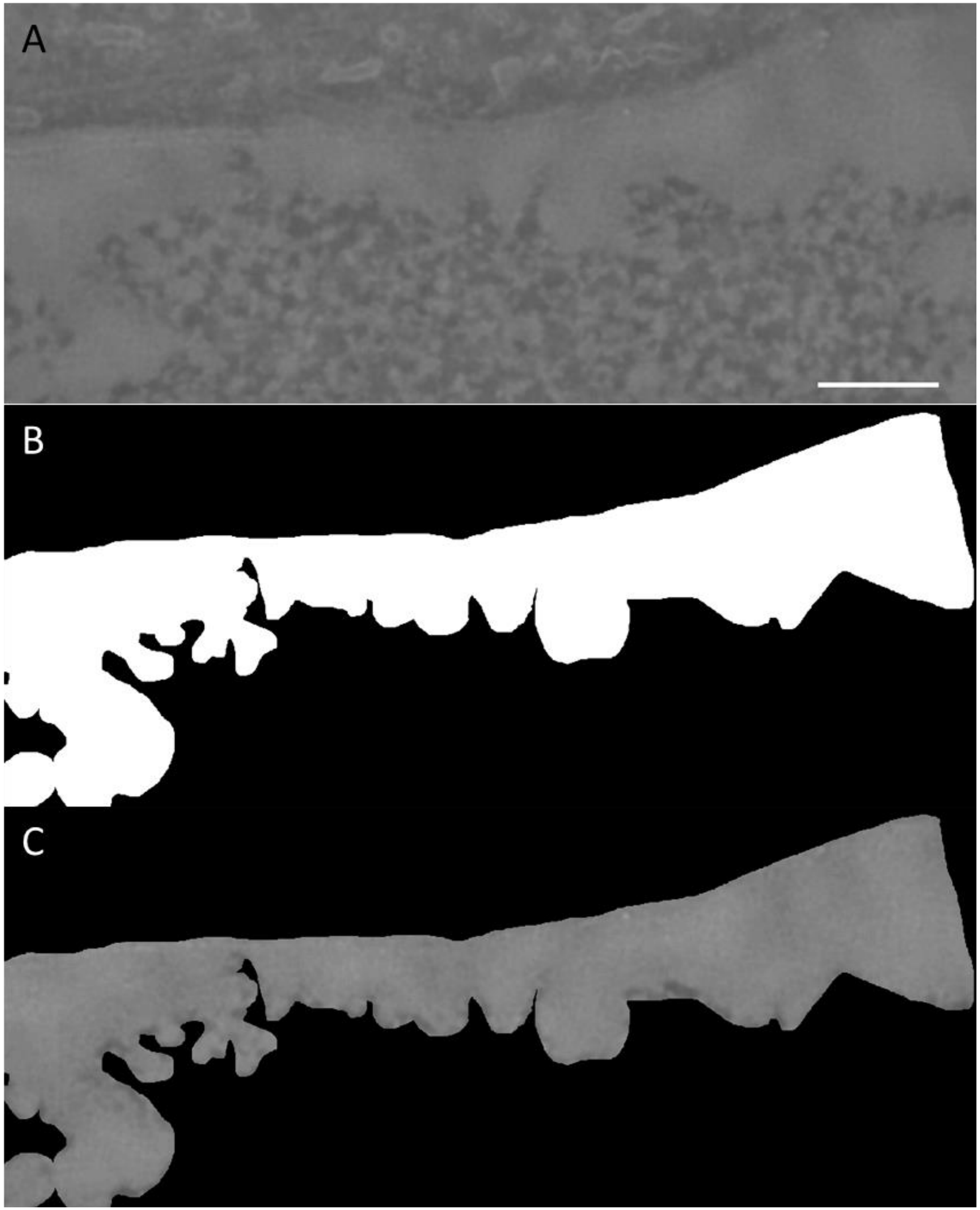
A
B
C

Supplemental Figure S7

- **Essential steps of fibre diameter analysis:**
- (A): inverted EMT image of a small subarea of the defined tomographic region. (B): Thresholding and binarisation after 3D median filtering. (C): Skeletisation of the fibre backbone. D: Visualization; Each yellow pixel (calculated centre of a fibre) contributes a distance value to nearest purple pixel (outline of fibre). Scale bar: 100 nm

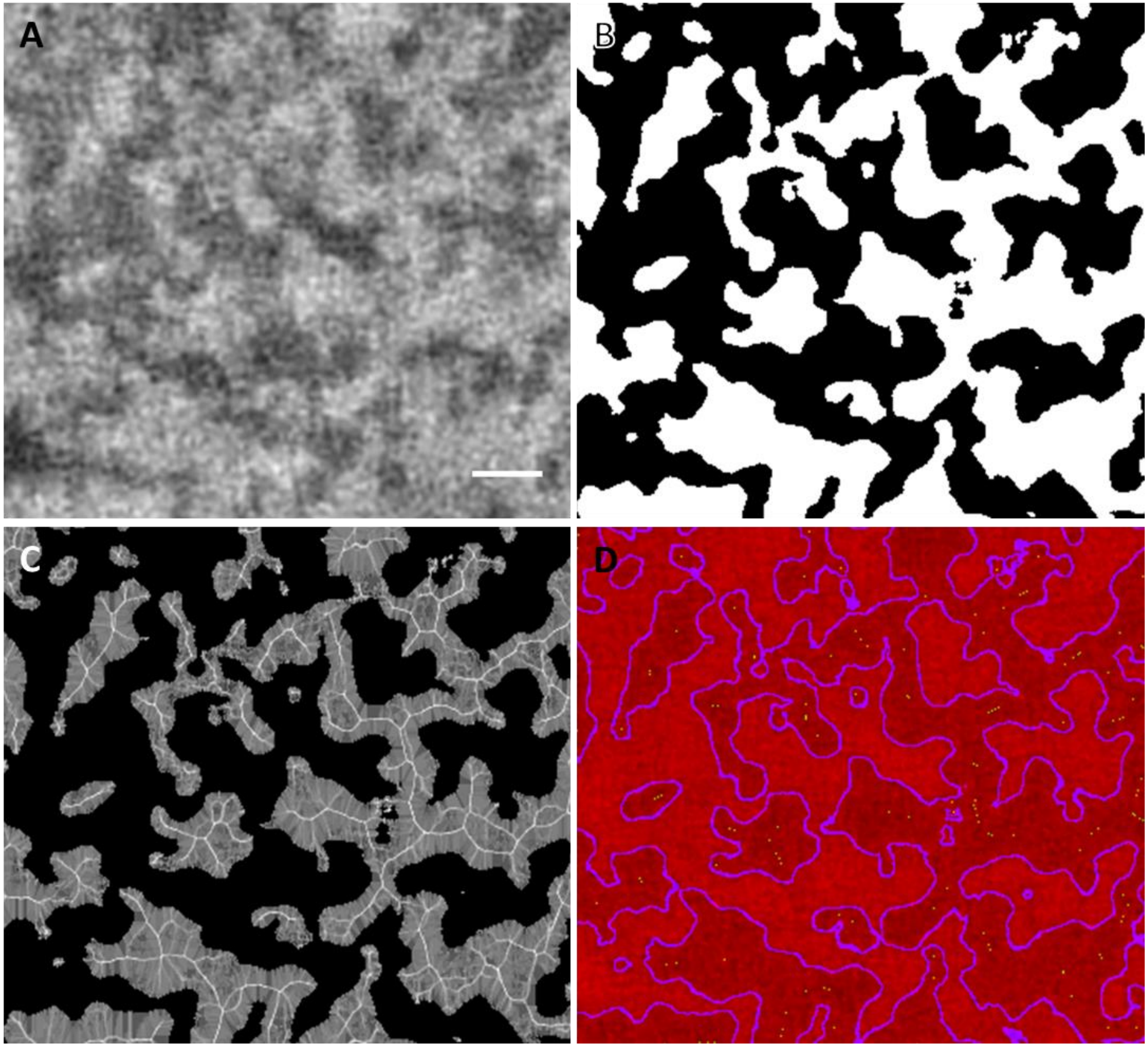
A
B
C
D

Supplementary Figure S8

- **Image of whole cell nucleus contrasted with RNA specific Terbium-Citrate stain:**
- TEM image of Terbium-Citrate (Tb) stained section (A, C) showing dense staining of nucleoli (nu) but lower contrast in areas of DNA damage (C-ions 1h, indicated by (B), green signal of 53BP1 and white arrow heads in C and D) as well as in HC areas (around nucleoli (C black arrow heads) and at nuclear envelope), which are characterised by bright DRAQ5 staining (magenta, B, D black arrow heads) indicating a high DNA content. Some areas show a higher Tb contrast (C arrows) correlating to low DNA density (D arrows). Scale bars: A&B 5 µm, C-F 1 µm

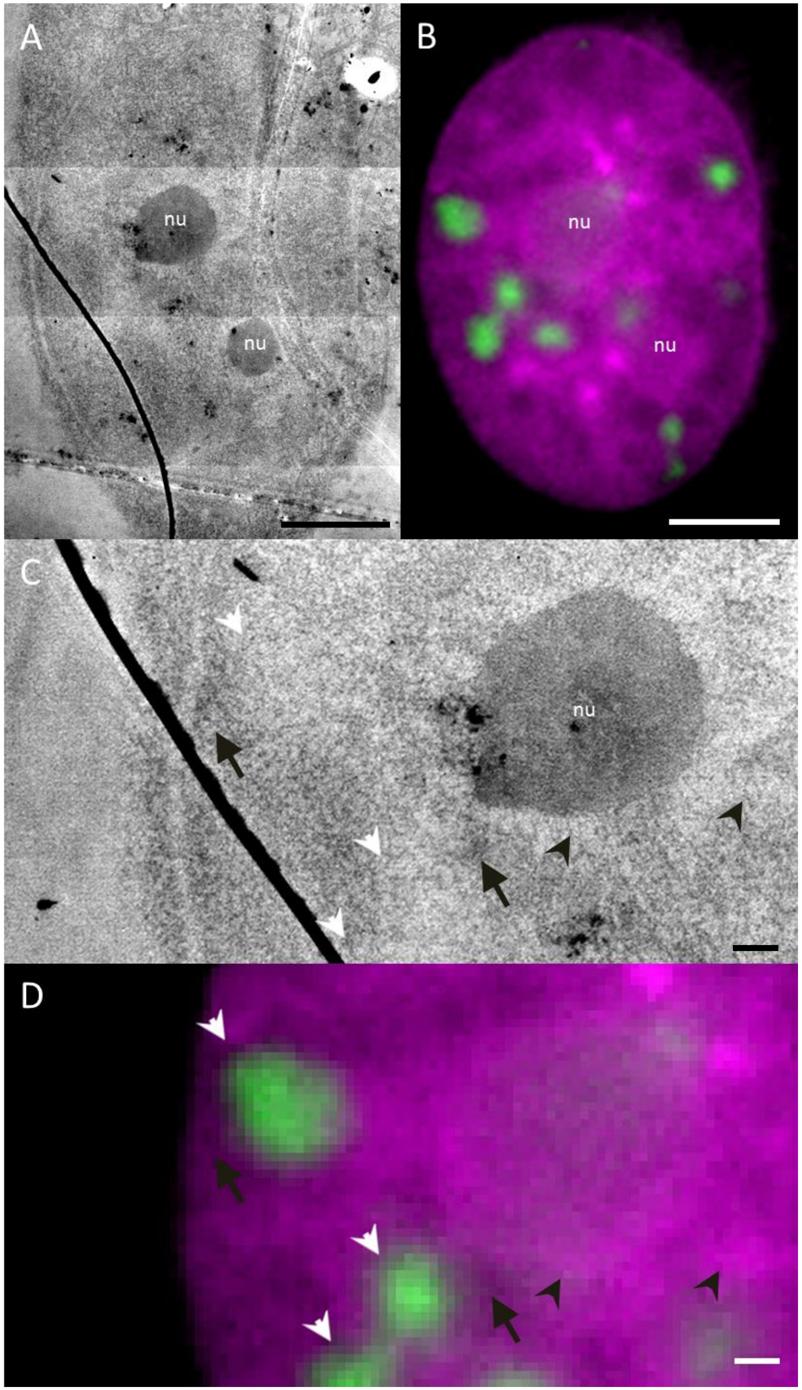
A
nu
nu
B
nu
nu
C
nu
D

Supplementary Figure S9

- **Terbium-Citrate stained section showing reduced RNA content at sites of 53BP1 IRIF 1h after irradiation with carbon ions**
- (A) Montage of EM image of Epon mounted sample showing whole cell of Figure 7. Additional LDAs at sites of damage indicated by 53BP1 accumulation in the corresponding FM image (B) are clearly visible. Scale bars: A&B 5 µm
- (C-F): Magnified CLEM images from (A/B) showing areas of LDA/IRIF. Scale bars: C-F 500 nm

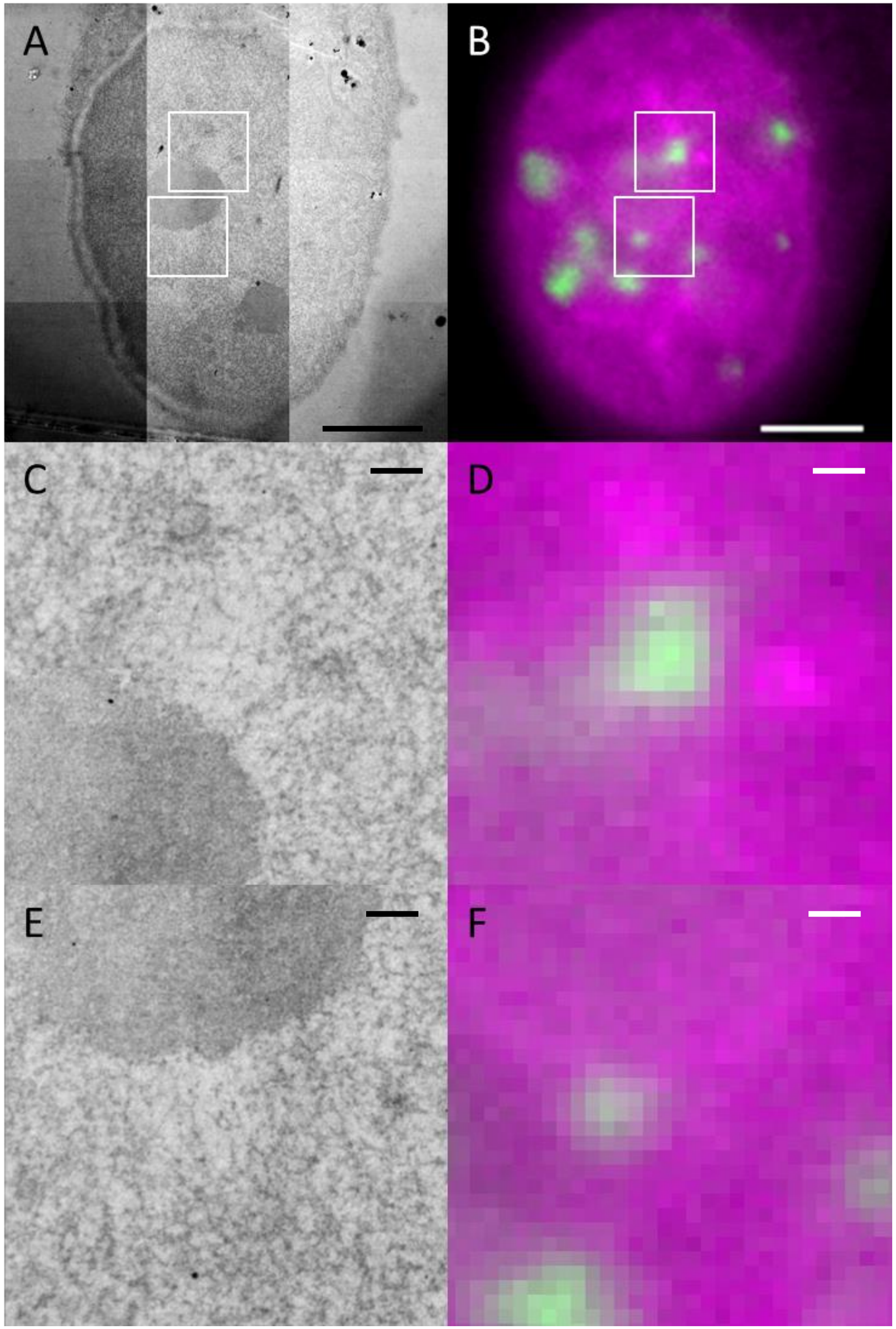

A
B
C
D
E
F

Supplementary Figure S10

- **Terbium-Citrate stained section showing reduced RNA content at sites of 53BP1 IRIF 5h after irradiation with iron ions in PFA-fixed/LR White embedded sample:**
- TEM image of Terbium-Citrate (Tb, A) stained section and FM image (B) of the same cell nucleus. Tb stained section shows less contrast in areas of DNA damage (indicated by (B), green signal of 53BP1). C-E show selected enlarged LDAs (white boxes in A and B). Scale bars: A&B 5 µm, C-E 500 nm

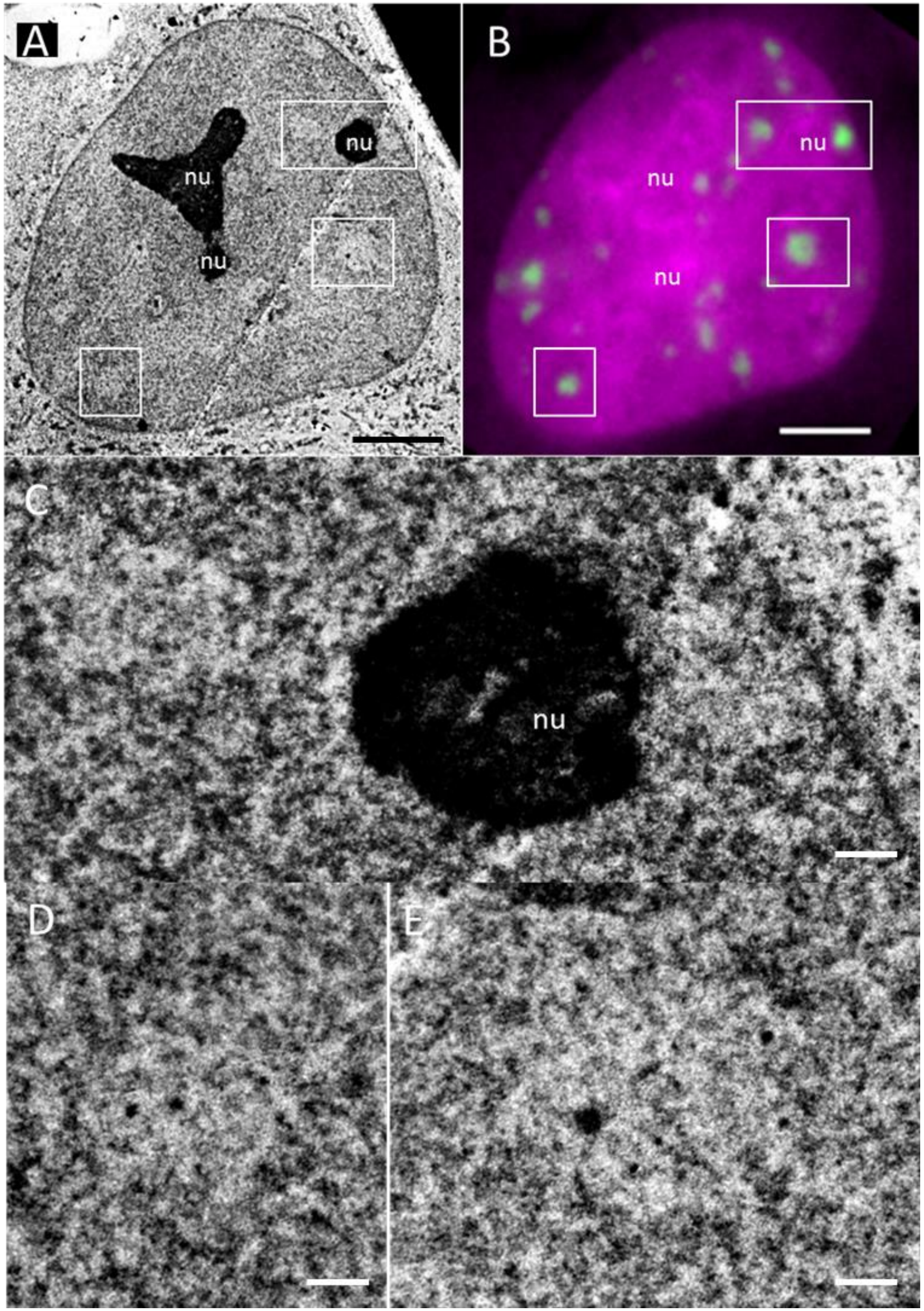
A
nu
nu
nu
B
nu
nu
nu
C
nu
D
E

Supplementary Figure S11

- **Sites of ion traversal show diminished RNA staining in fluorescence microscopy (FM):**
- **Left:** Mid section of a deconvoluted (Huygens essential SVI, The Netherlands) confocal image stack immunostained for 53BP1 (red) and for RNA (green, 500 nM Syto RNASelect Green, Molecular Probes INC, Eugene, USA). Upper panel: Overlay with indication of analysed 53BP1 RIF (yellow circle with radius of 1.4 µm); lower panel: separated channels. **Right:** Intensity profiles of radial projections at indicated sites of ion traversals indicating a loss of or otherwise diminished RNA signal (green) at 53BP1 IRIF (red). All scale bars: 5 µm.

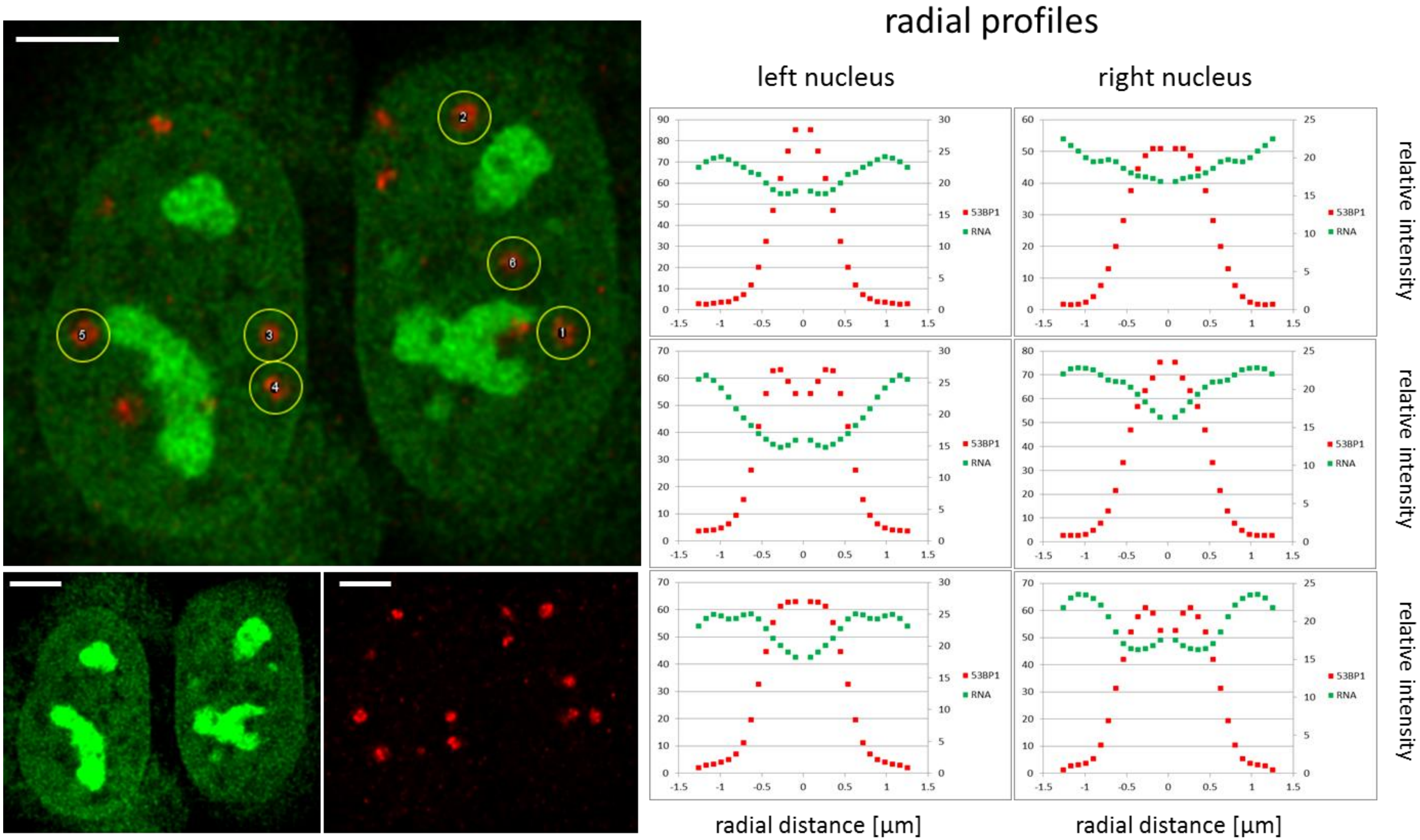
radial profiles
left nucleus
right nucleus
53BP1
RNA
relative intensity
radial distance [µm]
1
2
3
4
5
6
