# Supplementary figures and images for: Correlative Light and Electron Microscopy (CLEM) Analysis of Nuclear Reorganization Induced by Clustered DNA Damage Upon Charged Particle Irradiation
Source: Int J Mol Sci. 2020 Mar 11;21(6):1911. doi: 10.3390/ijms21061911 (PMC7139895; doi:10.3390/ijms21061911)

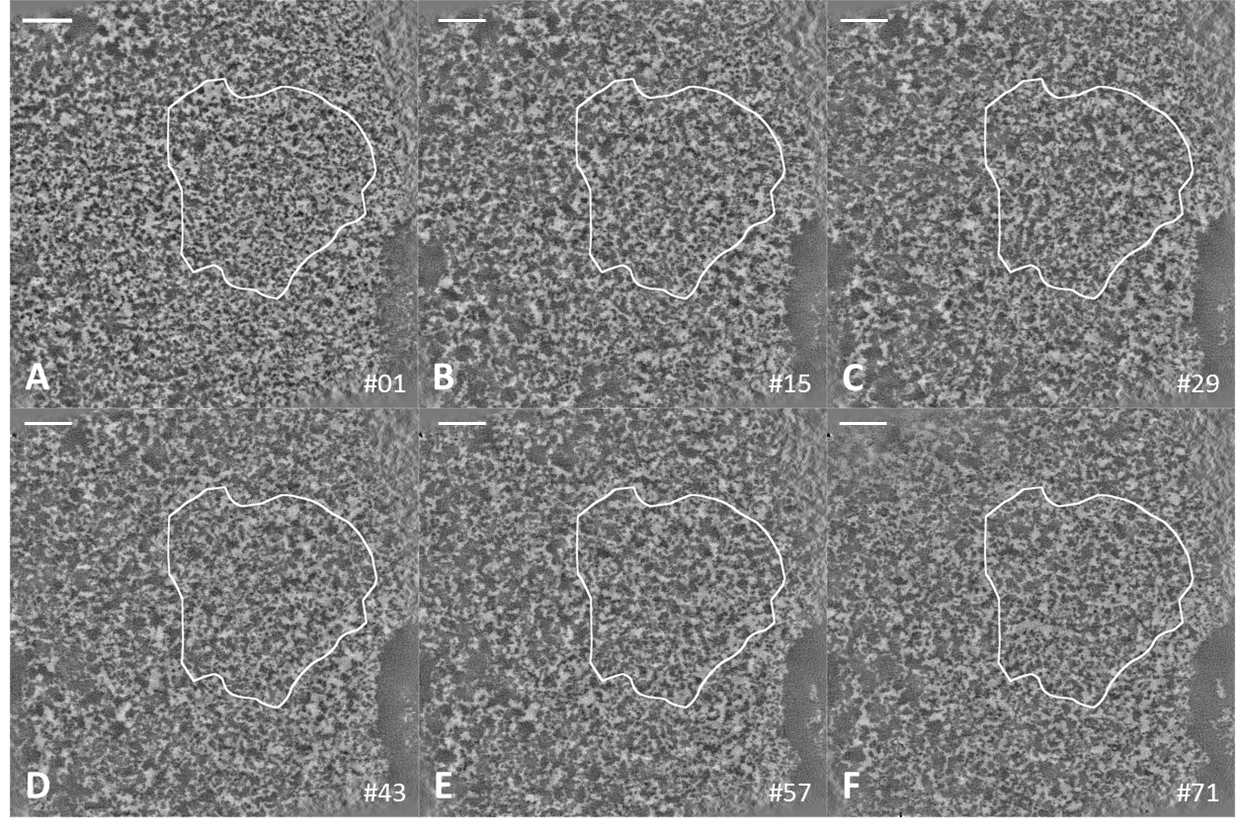

Supplement: Supplementary file 1 [file ijms-21-01911-s001.zip › SupplementalFigure_S5.tif]

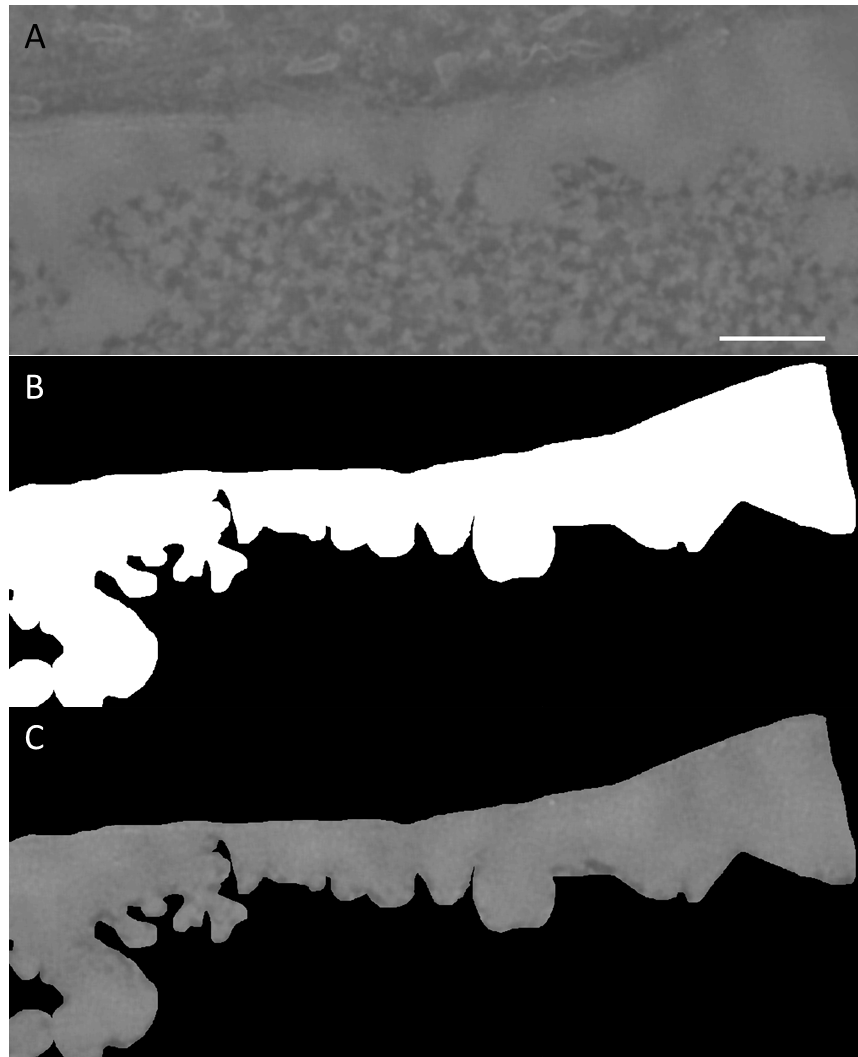

Supplement: Supplementary file 1 [file ijms-21-01911-s001.zip › SupplementalFigure_S6.tif]

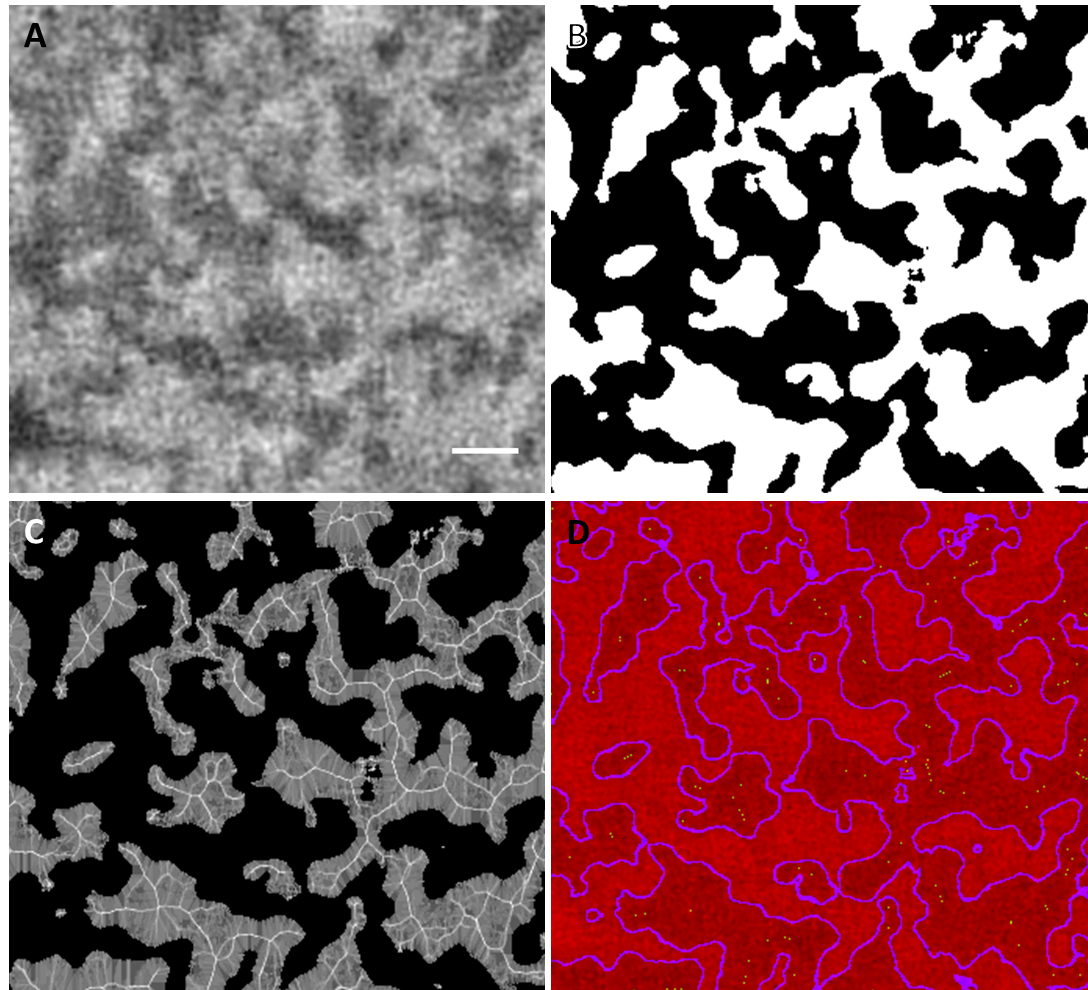

Supplement: Supplementary file 1 [file ijms-21-01911-s001.zip › SupplementalFigure_S7.tif]

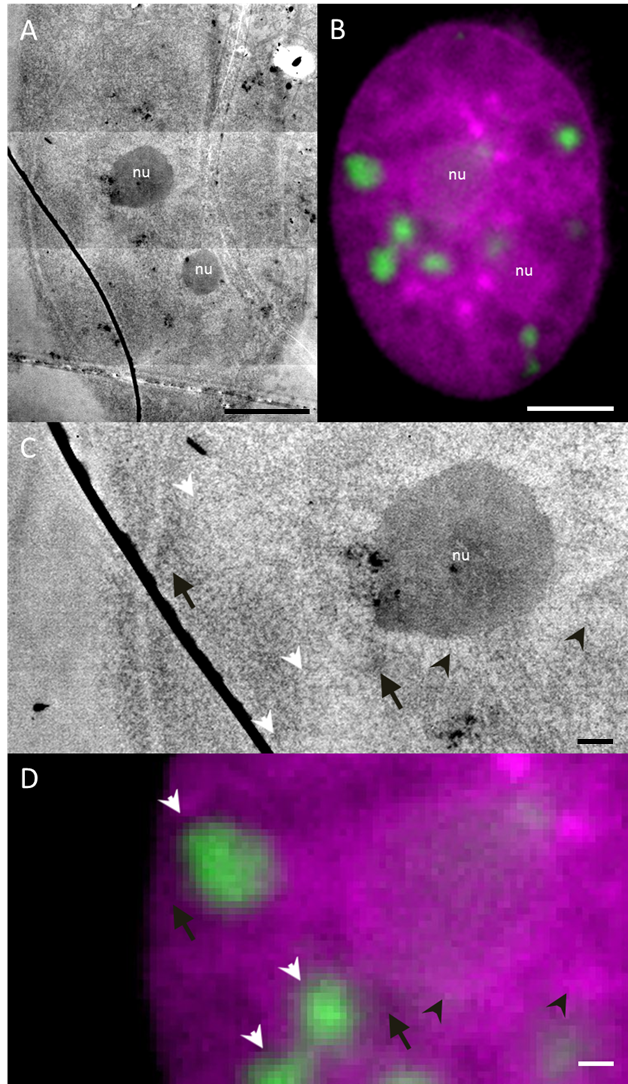

Supplement: Supplementary file 1 [file ijms-21-01911-s001.zip › SupplementalFigure_S8.tif]

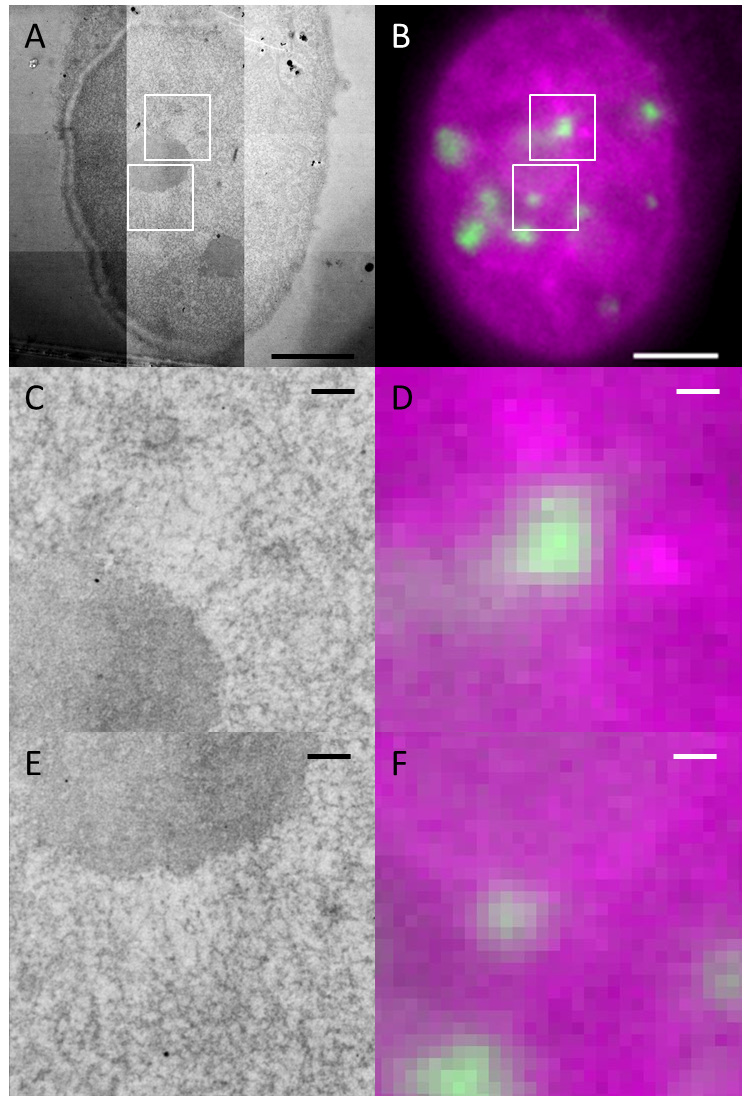

Supplement: Supplementary file 1 [file ijms-21-01911-s001.zip › SupplementalFigure_S9.tif]

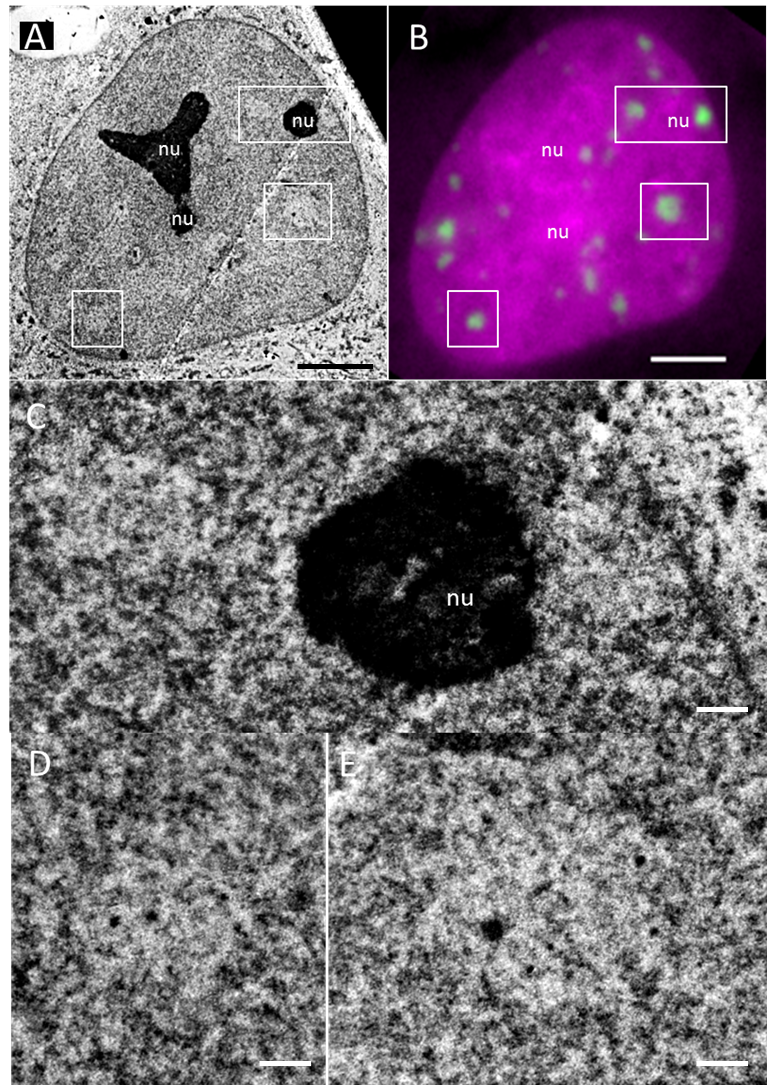

Supplement: Supplementary file 1 [file ijms-21-01911-s001.zip › SupplementalFigure_S10.tif]

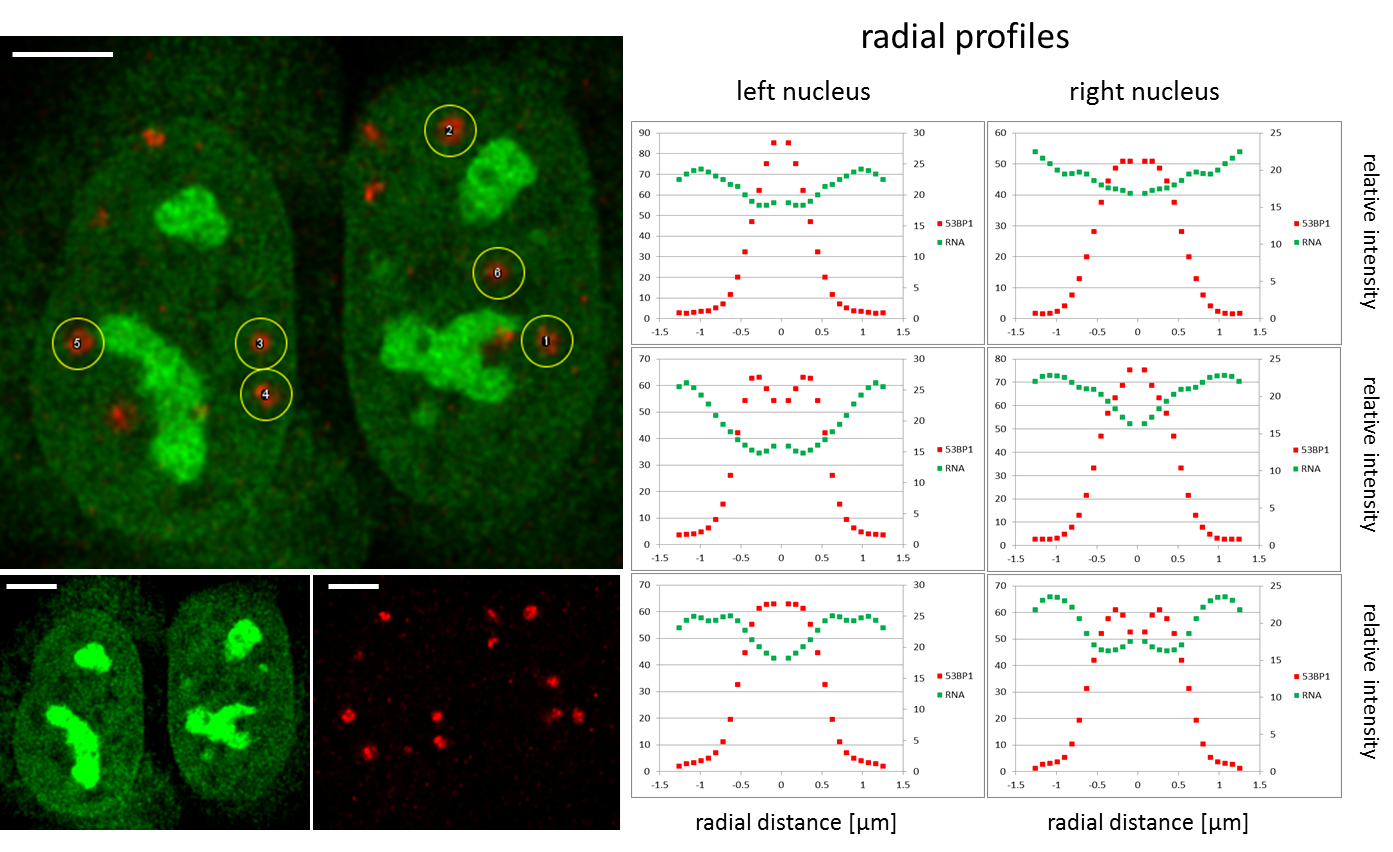

Supplement: Supplementary file 1 [file ijms-21-01911-s001.zip › SupplementalFigure_S11.tif]

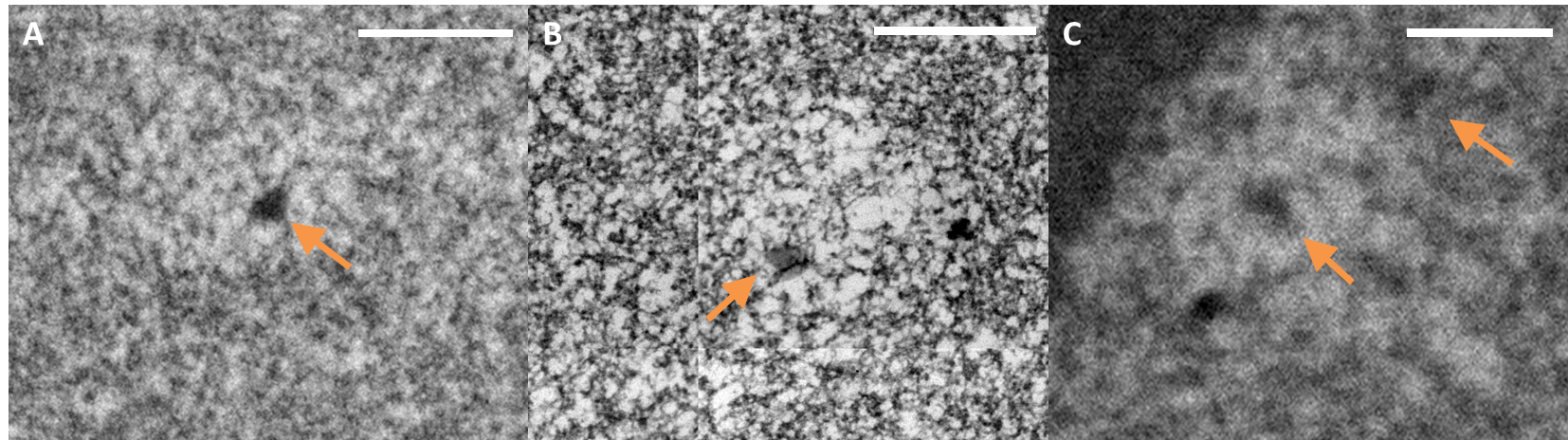

Supplement: Supplementary file 1 [file ijms-21-01911-s001.zip › SupplementalFigure_S1.tif]

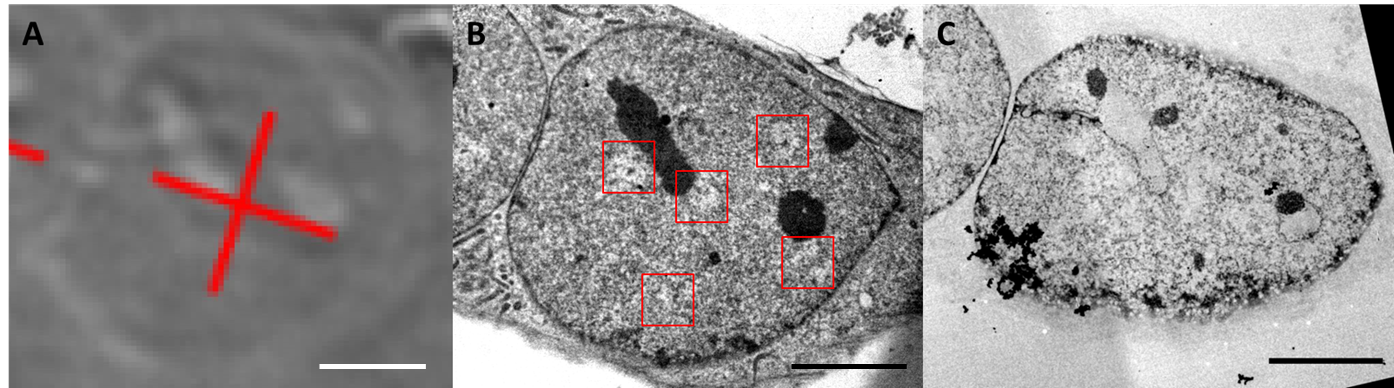

Supplement: Supplementary file 1 [file ijms-21-01911-s001.zip › SupplementalFigure_S2.tif]

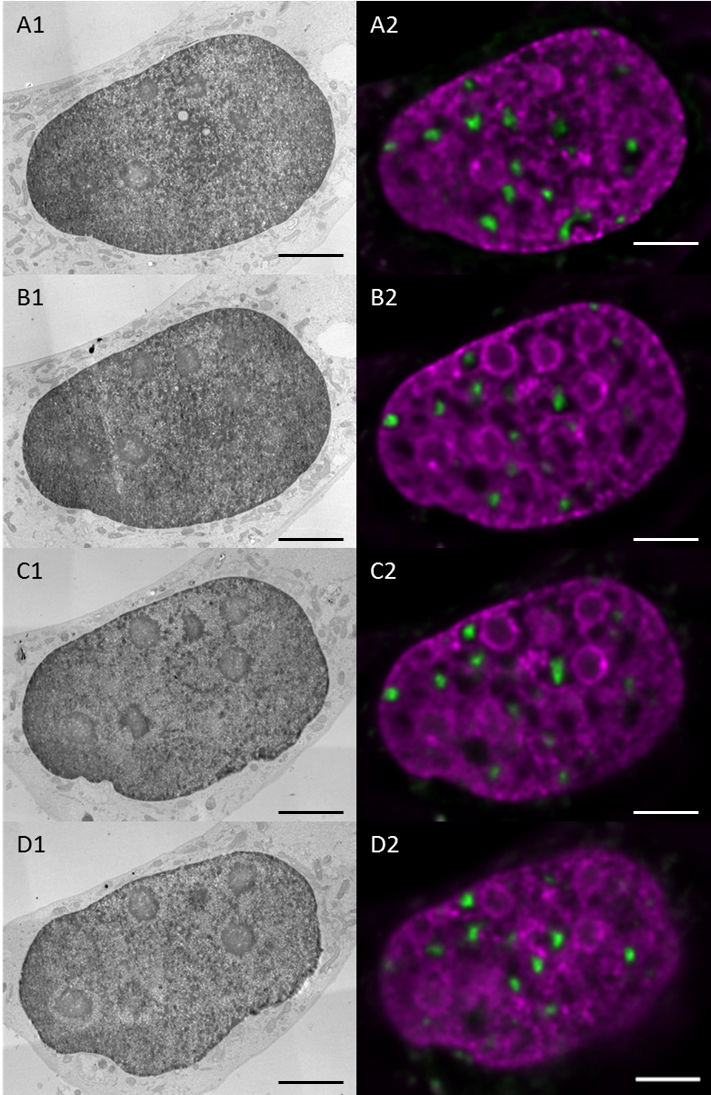

Supplement: Supplementary file 1 [file ijms-21-01911-s001.zip › SupplementalFigure_S3.tif]

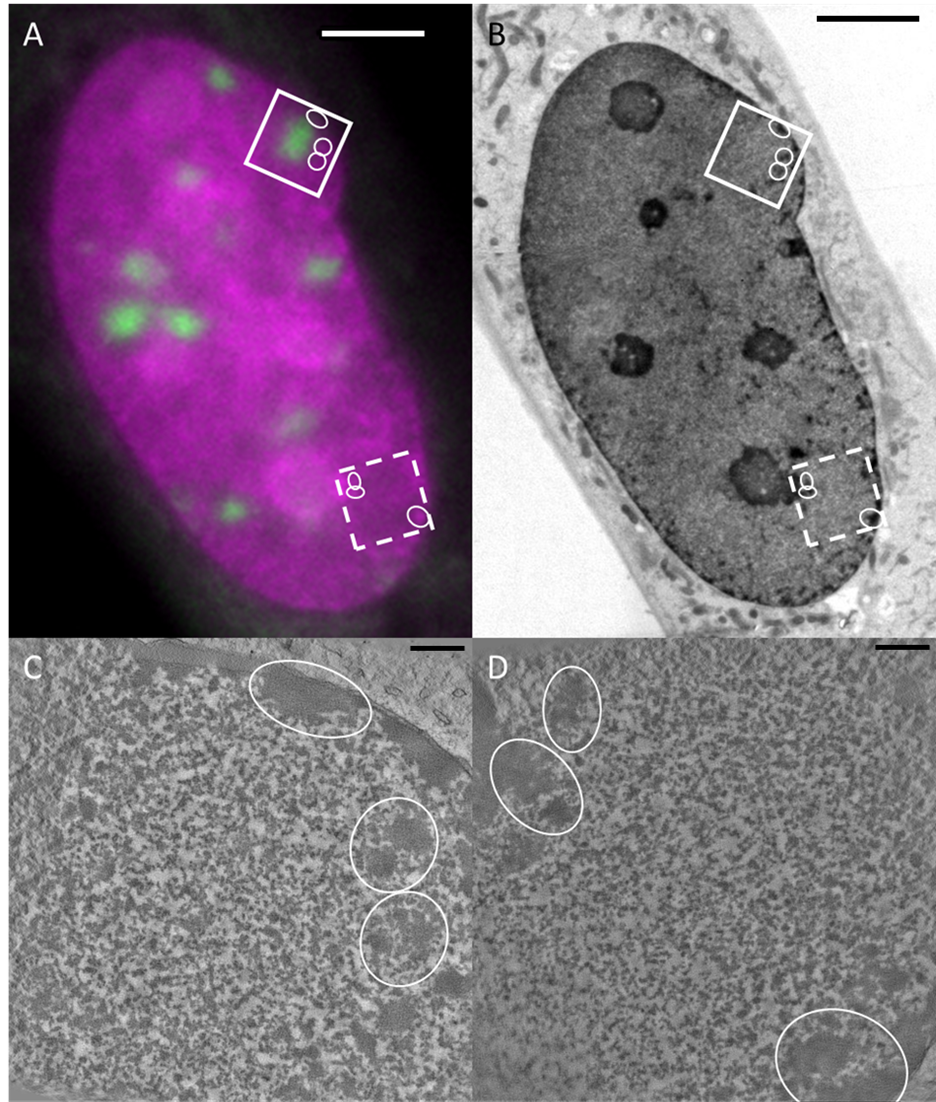

Supplement: Supplementary file 1 [file ijms-21-01911-s001.zip › SupplementalFigure_S4.tif]
